# Plasmid-encoded genes influence exosporium assembly and morphology in *Bacillus megaterium* QM B1551 spores

**DOI:** 10.1093/femsle/fnv147

**Published:** 2015-08-27

**Authors:** Julia Manetsberger, Elizabeth A. H. Hall, Graham Christie

**Affiliations:** Department of Chemical Engineering and Biotechnology, Institute of Biotechnology, University of Cambridge, Tennis Court Road, Cambridge CB2 1QT, UK

**Keywords:** *Bacillus*, spores, exosporium, indigenous plasmids, hairy nap

## Abstract

Spores of *Bacillus megaterium* QM B1551 are encased in a morphologically distinctive exosporium. We demonstrate here that genes encoded on the indigenous pBM500 and pBM600 plasmids are required for exosporium assembly and or stability in spores of this strain. Bioinformatic analyses identified genes encoding orthologues of the *B. cereus*-family exosporium nap and basal layer proteins within the *B. megaterium* genome. Transcriptional analyses, supported by electron and fluorescent microscopy, indicate that the pole-localized nap, identified here for the first time in *B. megaterium* QM B1551 spores, is comprised of the BclA1 protein. The role of the BxpB protein, which forms the basal layer of the exosporium in *B. cereus* spores, is less clear since spores of a null mutant strain display an apparently normal morphology. Retention of the localized nap in *bxpB* null spores suggests that *B. megaterium* employs an alternative mechanism to that used by *B. cereus* spores in anchoring the nap to the spore surface.

## Abstract (precis)

Essential components of the outermost shell that envelopes *Bacillus megaterium* spores are encoded on plasmid-located genes.

## Graphical abstract

**Graphical Abstract Figure.**
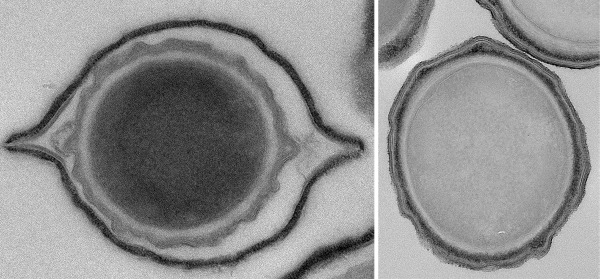
Essential components of the outermost shell that envelopes *Bacillus megaterium* spores are encoded on plasmid-located genes.

## INTRODUCTION

Bacterial members of the orders *Bacillales* and *Clostridiales* form environmentally resistant endospores in response to nutrient limitation. Spores of all species are encased in a proteinaceous outer shell, or coat, comprising upwards of 50 or more distinct proteins (Henriques and Moran 2007; McKenney, Driks and Eichenberger 2013). Much attention has been paid to the regulation and assembly of the spore coat, particularly in *Bacillus subtilis*, where significant progress has been made in elucidating the genetic networks and key morphogenetic proteins that control and underpin the assembly of this macromolecular structure (McKenney *et al.* 2010; McKenney and Eichenberger 2012). Spores of some species, however, including *B. cereus* and *B. megaterium*, contain an additional structure—the exosporium—which is morphologically distinct from the spore coat, and forms the outermost layer of the spore. Cryo-electron microscopy structural studies have revealed that the exosporium in *B. cereus* group members is formed of a crystalline lattice, or distinct layers of defined crystal types, that probably serve as environmental molecular sieves i.e. permitting ingress of small germinant molecules while excluding potentially harmful lytic enzymes and other macromolecules (Ball *et al.* 2008; Kailas *et al.* 2011).

While insights to the identity and assembly of a number of exosporium components have been achieved for *B. cereus* and *B. anthracis* spores (Todd *et al.* 2003; Redmond *et al.* 2004), little is known of the orthologous structure in *B. megaterium* spores. The latter species forms one of the major clades in the genus *Bacillus* and comprises a number of morphologically distinct strains. The best studied, particularly in the areas of spore germination and spore structure, is the QM B1551 strain, which carries ∼11% of its genome on seven indigenous plasmids (Rosso and Vary 2005; Eppinger *et al.* 2011). Despite also frequently carrying genetic information on plasmids, exosporium-encoding genes have not been identified as being plasmid borne in *B. cereus* family members. Accordingly, the purpose of this study was to examine whether plasmid-encoded genes influence the morphology of the *B. megaterium* QM B1551 exosporium.

## MATERIALS AND METHODS

### Bacterial strains and preparation of spores

*Bacillus megaterium* strains employed in this study (Table 1) were all isogenic with the QM B1551 strain. All strains were cultured on LB medium at 30°C, with antibiotics where appropriate (Table 1). Mutant strains were constructed by polyethylene glycol-mediated transformation of protoplasts. Transformant colonies that had undergone single or double crossover recombination events were isolated and verified by PCR, essentially as described previously (Gupta *et al.* 2013). Spores were prepared by nutrient exhaustion and purified as described previously (Gupta *et al.* 2013).

**Table 1.** *Bacillus megaterium* strains used in this study.

Strain	Relevant genotype*	Source
QM B1551	Wild-type strain	P.S. Vary
PV361	ΔpBM100, 200, 300, 400, 500, 600, 700	P.S. Vary
PV202	ΔpBM100, 600, 700	P.S. Vary
PV203	ΔpBM100, 300, 400, 600, 700	P.S. Vary
PV203-F	ΔpBM100, 300, 400, 500, 600	P.S. Vary
PV208	ΔpBM300, 400, 500, 600, 700	P.S. Vary
JM100	ΔpBM400, 700	This study
Null mutant strain		
JM411	Δ*bxpb*::Km^r^	This study
GFP/mCherry fusion strains		
JM412	*bxpB*::pVLG6 (*gfp*) Cm^r^	This study
JM413	*bclA1*::pVLG6 (*gfp*) Cm^r^	This study
JM414	*bclA2*::pVLG6 (*gfp*) Cm^r^	This study
JM415	*bclA1*::pVLG6 (*gfp*) Cm^r^	This study
JM416	pHT254-*bxpB*-*gfp* Cm^r^	This study
JM417	pHT254-*bclA1* (codons 1-159)*-gfp* Cm^r^	This study
JM418^†^	pHT254-*bclA1* (codons 1-159)*-gfp* Cm^r^	This study
JM419	*Alr*::pVLG6 (*gfp*) Cm^r^	This study
JM708	*sleL*::pVLG6 (*gfp*) Cm^r^	Manetsberger, Hall and Christie (2014)
JM709	*cotX*::pVLG7 (*mCherry*) Cm^r^	Manetsberger, Hall and Christie (2014)

*Abbreviations for antibiotics: Km^r^, kanamycin resistance (5 μg mL^−1^); Cm^r^, chloramphenicol resistance (5 μg mL^−1^).

^†^This strain was constructed in the Δ*bxpb*::Km^r^ background.

### Molecular biology procedures

Transcriptional analysis from loci of interest was examined by RT-PCR, using gene-specific primers designed to amplify ∼400 bp DNA fragments (Ramirez-Peralta *et al.* 2013). *Bacillus megaterium* strains bearing C-terminal GFP and mCherry fusions to proteins of interest were constructed as described previously (Manetsberger, Hall and Christie 2014). A plasmid designed to facilitate the construction of a strain bearing a truncated version of BclA1 (BMQ_pBM50077), in which the predicted N-terminal domain of the protein was fused at the C-terminus to GFP (i.e. omitting the collagen-like region of the protein) was prepared by cloning a 477 bp DNA fragment, encompassing codons 1–159 of the *bclA1* gene, into pVLG6. Additionally, a number of strains were prepared in which genes of interest were placed under the control of an IPTG-inducible promoter on the episomal pHT254 plasmid. Essentially, PCR was used to prepare DNA fragments that encoded either the entire open reading frame (minus stop codons) or a defined fragment of the gene of interest, with a *gfp* amplicon fused in-frame at the 3^′^ end. PCR amplicons were ligated with linearized pHT254, and the resultant plasmids used to transform *B. megaterium* to chloramphenicol resistance. Transformant clones were verified by PCR, and inducible protein expression initiated in sporulating cultures (upon entry to stationary phase) via addition of 1 mM IPTG.

A *B. megaterium bxpB* (BMQ_pBM60048) null mutant strain was created via allelic exchange with a kanamycin resistance cassette flanked by 200-bp fragments from the 5^′^ and 3^′^ ends of the *bxpB* ORF, employing procedures described previously (Gupta *et al.* 2013). Sequence information for all oligonucleotides used in this work is available upon request. Cloning and propagation of plasmids was achieved using *Escherichia coli* DH5α (NEB, Hitchin, UK), cultured typically in LB medium supplemented with 50 μg mL^−1^ carbenicillin.

### Microscopy

Fluorescence and thin-section transmission electron microscopy (TEM) analyses were conducted as described previously (Manetsberger, Hall and Christie 2014). Negatively stained intact spores were imaged using a Philips CM100 transmission electron microscope operated at an accelerating voltage of 100 kV. Micrographs were collected at a size of 1024 × 1024 pixels using a Gatan Multiscan 794 1k × 1k CCD camera and analysed with Gatan Digital Micrograph software (DM, Gatan Inc.).

## RESULTS

### Electron microscopy analysis of *B. megaterium* QM B1551 spores

**Figure 1.**
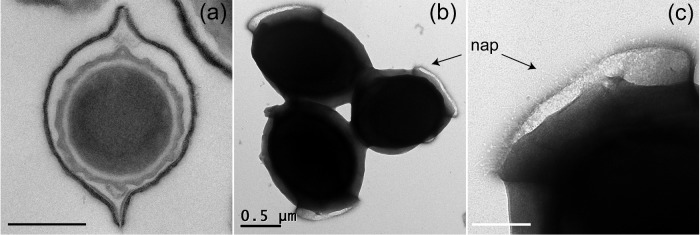
Electron microscopy analysis of *B. megaterium* QM B1551 spores. Transmission electron micrographs of (**a**) sectioned, and (**b**) intact wild-type spores. The localized hair-like nap visible as a diffuse layer in (b) is shown in close-up in (**c**). Scale bars represent 500 nm (a and b) and 200 nm (c), respectively.

TEM was used to investigate the ultrastructure of *B. megaterium* QM B1551 spores. Thin-sectioned TEM images revealed the typical *B. megaterium* QM B1551 spore architecture i.e. a centralized spore core (protoplast) surrounded by the electron translucent peptidoglycan cortex, with contiguous layers of lamellar coat material and then the outermost ‘walnut-like’ exosporium (Fig. 1a) (Beaman, Pankratz and Gerhardt 1972). The extended poles of the exosporium are revealed in images of negatively stained intact spores to result from a flattened rim of exosporium material that surrounds the densely stained inner-spore integuments (Fig. 1b). Analysis of intact spores revealed a novel morphological feature not previously associated with *B. megaterium* QM B1551 spores. Discernible filament-like structures, which we assume to be analogous to the hair-like nap evident on *B. cereus* family spores, are localized to a projected ring or sheath of exosporium material located at one of the spore poles (Fig. 1b and c). To our knowledge, these images confer the first evidence for the presence of a nap on *B. megaterium* QM B1551 spores.

### Influence of plasmids on *B. megaterium* QM B1551 spore morphology

**Figure 2.**
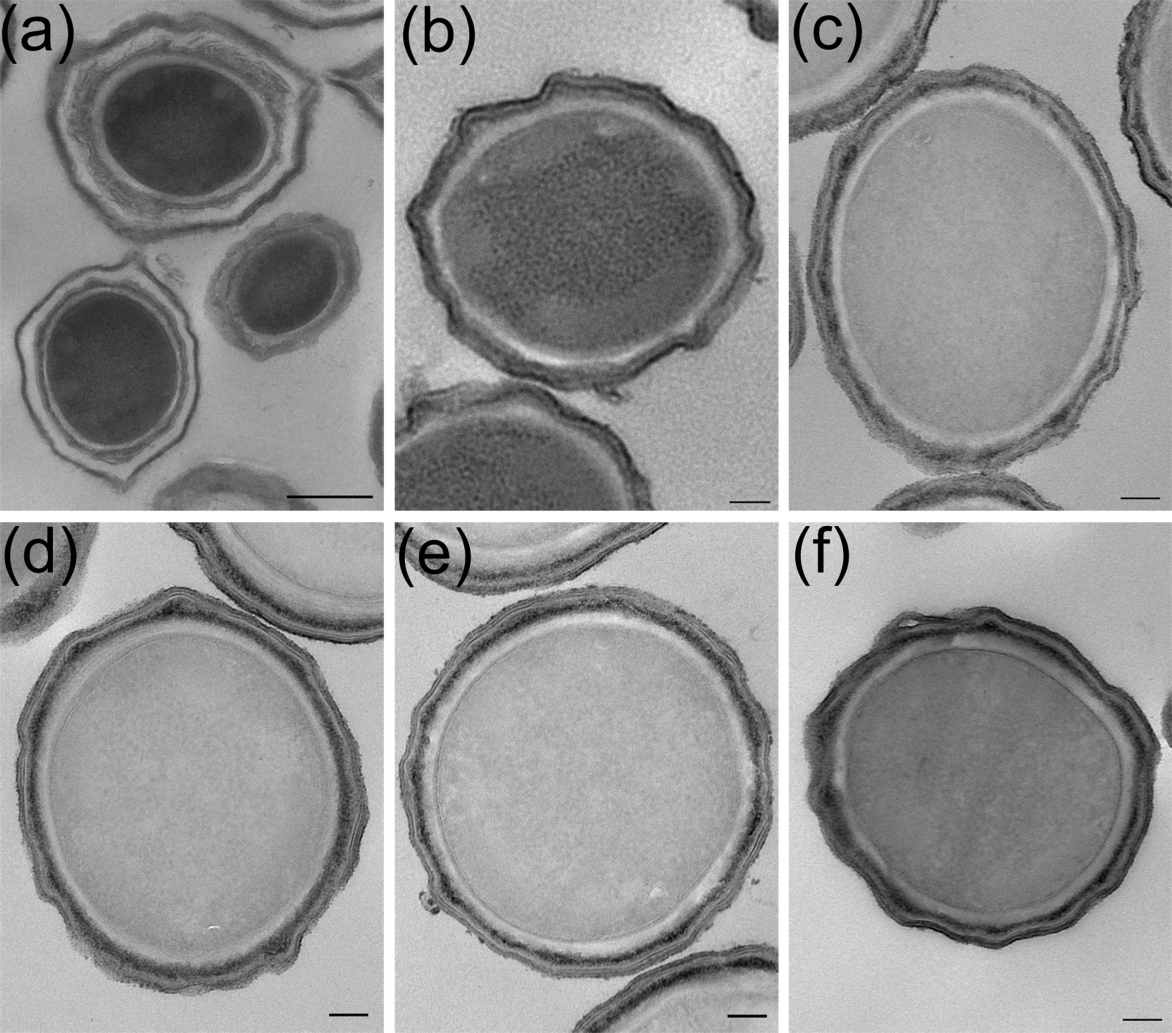
TEMs of *B. megaterium* QM B1551 plasmid-variant strains. (**a**) JM100 [1,2,3,5,6], (**b**) PV203-F [2,7], (**c**) PV202 [2,3,4,5], (**d**) PV203 [2,5], (**e**) PV208 [1,2] and (**f**) PV361. Numbers in square brackets refer to plasmids present in each strain, where 1 = pBM100, etc. Scale bars represent 500 nm (a) and 100 nm (b–f), respectively.

*Bacillus megaterium* QM B1551 carries seven indigenous plasmids that range in size from the 5.5 kb pBM100 to the 165 kb pBM700. In order to assess the impact of plasmid-encoded genes on spore morphology, we obtained a number of plasmid-cured strains that had been characterized previously for plasmid content by plasmid purification and gel electrophoresis (Stevenson, Lach and Vary 1993). We reexamined these strains, and isolates from our own laboratory, using a more reliable PCR-based method that utilized defined plasmid-specific oligonucleotides (Table 1 and Fig. S1, Supporting Information). Having established plasmid profiles for the different strains, TEM was then employed to characterize spore morphology. These analyses revealed that spores of the PV361 strain, which lacks all seven plasmids, lack an exosporium, although the coat and cortex appear normal (Fig. 2). In support of the latter, resistance to wet heat and lysozyme in all mutant strains was comparable to wild-type spores (data not shown). Wild-type spores are, however, considerably more hydrophobic and adhere to a range of materials much more tightly than exosporium-less PV361 spores, indicating that at least one function of the *B. megaterium* exosporium concerns the attachment of spores to surfaces (Ke Xu Zhou, unpublished results). Similarly, several other strains were observed to have lost their exosporium (PV202, PV203, PV203-F, PV208), while retaining apparently normal underlying layers. A common feature of these strains is that they lack the pBM600 plasmid. The collective presence of plasmids pBM200 through to pBM500 (strain PV202) is evidently insufficient to support exosporium development, whereas pBM400 and pBM700 are dispensable (strain JM100). Hence, based on the relatively limited analysis of mutant strains described above, it seems that essential components of the *B. megaterium* QM B1551 exosporium are encoded on plasmid pBM600. Additionally, since we have yet to isolate a strain that lacks pBM500 but retains pBM600 we cannot rule out an essential role for pBM500 in exosporium formation.

### Plasmid-associated exosporium genes

Homology searches were conducted against the *B. megaterium* QM B1551 genome using the NCBI Translated BLAST *tblastn* program using established exosporium proteins from *B. cereus* and *B. anthracis* as seeds (Sylvestre, Couture-Tosi and Mock 2002; Todd *et al.* 2003; Redmond *et al.* 2004). Of the four hits that were returned, two orthologues of the *B. cereus*/*anthracis* hairy-nap protein, BclA, were identified as being encoded on plasmid pBM500 (BMQ_pBM50077 and BMQ_pBM50081; Fig. S2, Supporting Information). BMQ_pBM50077 encodes a 954-amino-acid protein with an estimated molecular mass of 87 kDa. It incorporates 220 GXT repeats in its sequence (where X is normally I or A), characteristic for collagen-like proteins, and has been assigned the name BclA1. BMQ_pBM50081 has a predicted molecular weight of 44 kDa, including 40 GXX repeats, and has been assigned the name BclA2. In addition to the characteristic collagen-like regions, both proteins are predicted to have globular N- and C-terminal domains. The N-terminal domains appear to differ markedly from the orthologous domain in *B. anthracis* BclA i.e. the *B. megaterium* BclA1 N-terminal domain comprises 159 residues, compared to 38 residues in *B. anthracis* BclA. Furthermore, neither N-terminal domain appears to contain the sequence motif required for incorporation of the orthologous BclA/BclB proteins into the *B. anthracis* exosporium (Thompson and Stewart 2008; Tan and Turnbough 2010) (Fig. S3, Supporting Information). In contrast, the C-terminal domains both comprise ∼135 residues (134 in *B. anthracis*) and share 17% sequence identity (30% similarity).

Homology searches yielded also an orthologue of the *B. anthracis* BxpB exosporium basal layer protein (ExsFA in *B. cereus*), which is encoded on plasmid pBM600 at locus BMQ_pBM60048 (Fig. S2, Supporting Information). *Bacillus megaterium* BxpB has a predicted molecular mass of 17 kDa, and shares 26% sequence identity (45% similarity) at the amino acid level with *B. anthracis* BxpB. The only other putative exosporium protein identified from *tblastn* searches as being encoded within the *B. megaterium* QM B1551 genome is an orthologue of alanine racemase (Alr), which is encoded on the chromosome (BMQ_0226) and is predicted to share 39% sequence identity with the *B. anthracis* protein.

### Expression and localization of *B. megaterium* BclA1, BclA2 and BxpB

**Figure 3.**
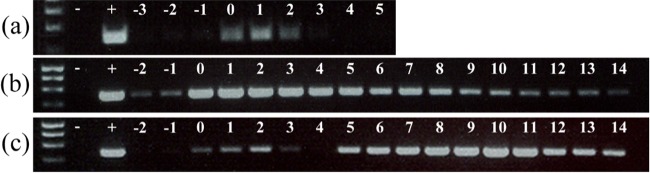
Transcription of (**a**) bclA2, (**b**) bclA1 and (**c**) bxpB during sporulation of *B. megaterium* QM B1551. RT-PCR was conducted on samples collected at hourly intervals preceding and during sporulation. Key: – and +, RT-PCR controls; numbers denote time (h) preceding and post entry to stationary phase (0).

Reverse transcription polymerase chain reaction (RT-PCR) was conducted on cDNA derived from cellular samples collected throughout sporulation in order to examine transcription from the *bclA1*, *bclA2* and *bxpB* loci (Fig. 3). These analyses were compromised to an extent by the relative asynchronicity of sporulation in SNB medium; however, the low abundance of PCR products associated with the *bclA2* locus indicates that the BclA2 protein is expressed at only very low levels.

Expression of the longer collagen-like protein, BclA1, was more readily detected throughout the course of sporulation (PCR products of low abundance detected prior to entry to stationary phase presumably reflect asynchronous sporulation). Based on the abundance of PCR products, expression of BclA1 appears highest during the early stages of sporulation, an observation that is consistent with a potential σ^E^ consensus sequence identifiable upstream of the *bclA1* ORF (data not shown). RT-PCR products indicate that the BxpB protein is expressed predominantly during the mid to later stages of sporulation, although less abundant PCR products are evident early in sporulation also. Sequence analysis revealed poorly conserved potential σ^E^ and σ^K^ promoter sequences upstream of the predicted *bxpB* start codon (data not shown), which is predicted to initiate with a TTG codon as opposed to ATG.

**Figure 4.**
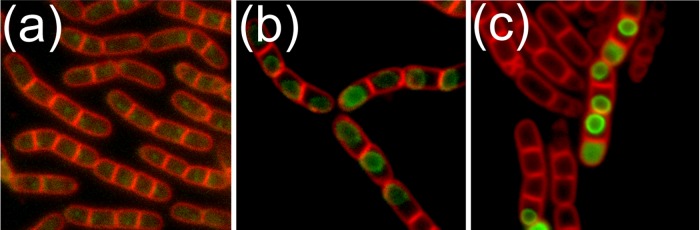
Fluorescence micrographs showing expression and localization of putative *B. megaterium* exosporium basal layer and nap proteins. (**a**) Diffuse mother cell localized fluorescence associated with expression of BxpB-GFP. (**b**) Localization of BclA1-GFP to the forespore during sporulation. (**c**) C-terminally truncated BclA1 (residues 1–159) retains the ability to localize to the developing forespore.

Expression and localization of the putative exosporium basal layer and nap proteins was examined further by creating strains with 3^′^ *gfp* fusions to *bxpB*, *bclA1* and *bclA2*. Only faint fluorescence associated with BxpB-GFP was observed in the mother cell during sporulation of strain JM412, with no fluorescence detectable at all in the mature spores (Fig. 4a). Similarly, expression of BxpB-GFP under control of the inducible P*grac*100 promoter failed to enhance fluorescence levels, precluding the localization of the protein in the spore. Expression of BclA1-GFP during sporulation was associated with diffuse green fluorescence in the mother cell, followed by the localization and formation of a bright ring of fluorescence around the developing forespore (Fig. 4b). However, fluorescence diminished upon the release of the spore from mother cells, and the fusion protein could no longer be detected with certainty on the spore surface. Similarly, fluorescence associated with the localized nap feature described above could not be discerned above background levels by fluorescence microscopy. Analysis of strain JM417, engineered to express only the predicted N-terminal domain of the BclA1 protein as a GFP fusion under control of the inducible P*grac*100 promoter, revealed a similar pattern of localization around the developing forespore (Fig. 4c). This domain, like the orthologous domain in *B. anthracis* spores (Tan and Turnbough 2010), is presumably responsible for localizing the nap protein to the spore surface despite sharing limited, if any, similarity to the targeting motif that has been characterized in the *B. anthracis* protein. However, as with the full-length fusion protein, fluorescence was lost upon maturation of the spore.

RT-PCR experiments also indicated that *B. megaterium* Alr is expressed throughout sporulation (data not shown). However, a strain engineered to express a C-terminal GFP fusion did not show any appreciable fluorescence, and no further analyses were conducted.

### BxpB null mutant spores

**Figure 5.**
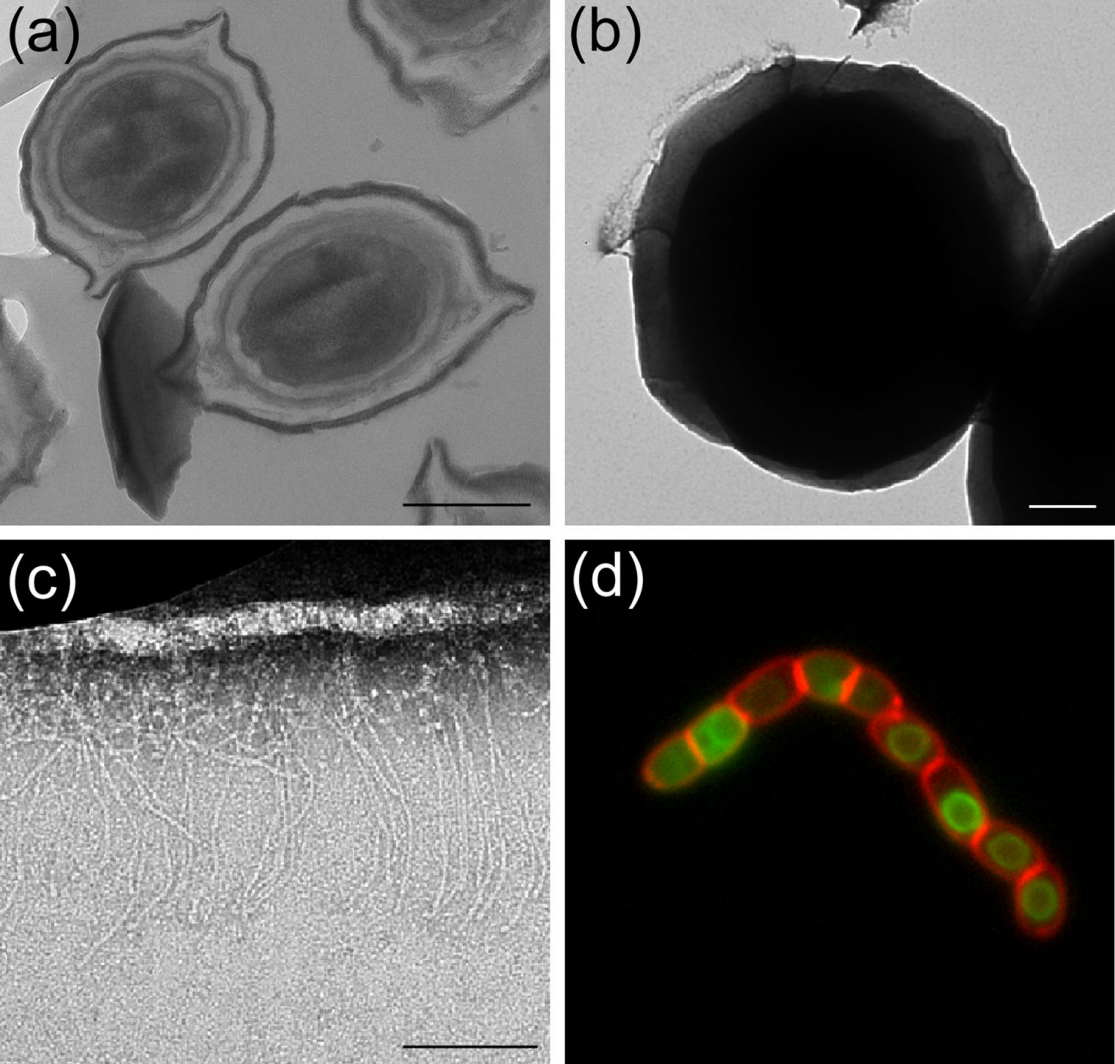
EM and fluorescence microscopy analysis of *B. megaterium* bxpB spores. (**a**) Thin-section TEM, showing spores with apparently normal morphology, although an exosporium fragment is also visible. (**b**) Negatively stained intact ΔbxpB spores. The localized nap is magnified in (**c**), where the length of the fibres is consistent with the predicted length of the collagen-like domain of BclA1 (∼190 nm). (**d**) Fluorescence micrograph showing that C-terminally truncated BclA1 localizes to the forespore during sporulation despite the absence of BxpB. Scale bars represent (a) 500, (b) 200 and (c) 100 nm.

The *bxpB* gene was disrupted by allelic exchange with an antibiotic resistance cassette in order to examine the role of the BxpB protein in exosporium assembly. The orthologous protein forms the exosporium basal layer in *B. cereus*/*anthracis* spores and interacts with BclA and BclB to anchor the nap proteins to the spore surface (Sylvestre, Couture-Tosi and Mock 2005; Thompson *et al.* 2011). However, TEM analyses revealed apparently normal looking spore architecture in *B. megaterium bxpB* spores, including an intact exosporium (Fig. 5a). Unexpectedly, TEM analysis of negatively stained *ΔbxpB* spores revealed the retention of the localized nap on mutant spores (Fig. 5b and c). Similarly, fluorescence microscopy revealed a similar pattern of localization of the truncated BclA1-GFP protein in *bxpB* spores to that observed in the wild-type background (Fig. 5d). Fluorescence microscopy also revealed that deletion of *bxpB* has no effect on the assembly of the SleL inner spore coat protein (Fig. S4, Supporting Information). The putative outer coat/exosporium protein, CotX2, was also observed to localize around *bxpB* spores in a similar manner to that observed in the wild-type background (Fig. S4, Supporting Information). In this case, however, shards of fluorescent material were observed to detach from the surface of some spores within the population, indicating perhaps that the loss of BxpB has compromised the structural integrity of the exosporium.

## DISCUSSION

The present study has revealed several new insights to the structure and assembly of the outermost layers of *B. megaterium* QM B1551 spores. First, we provide evidence that essential exosporium gene products are plasmid encoded. To our knowledge, this is the first demonstration of such an association between exosporium formation and plasmid-borne genes within the *Bacilli*. A common genotype in all exosporium-less strains examined in the course of this work was the absence of the 67-kb pBM500 and 100-kb pBM600 plasmids, indicating that proteins crucial to exosporium assembly, structural integrity and/or stability are encoded on these plasmids. Neither plasmid is essential for viability, so presumably selective pressure in the environmental niche originally inhabited by this strain outweighs the metabolic burden associated with plasmid maintenance.

Second, TEM analyses of negatively stained intact spores revealed the presence of a hair-like nap, localized to one of the spore poles. Bioinformatic, transcriptional and fluorescence/electron microscopy analyses indicate that the nap is composed of fibres formed by the BclA1 protein, which is encoded on pBM500, one of the plasmids essential for exosporium assembly. Unfortunately, we have been unable to corroborate this hypothesis by examination of BclA1 null mutant spores since several attempts aimed at constructing this strain have failed. However, despite lacking the consensus sequence identified within the orthologous *B. anthracis* protein, we have been able to demonstrate that the N-terminal domain of *B. megaterium* BclA1 is involved in localizing the nap to the developing forespore. Surprisingly, the protein appears to localize around the entire surface of the developing spore, rather than being localized to one of the poles. However, subsequent loss of fluorescence during spore maturation may reflect detachment of the protein from the bulk of the spore surface, with only pole-localized BclA1 fibres remaining. Whether the nap-associated pole represents an analogous structure to the ‘bottle-cap’ from which *B. anthracis* cells emerge during spore germination (Steichen, Kearney and Turnbough 2007) remains to be ascertained.

Finally, the role of the BxpB protein—a key structural component in the exosporium of members of the *B. cereus* family—has been examined in this work. The orthologous *B. megaterium* protein is encoded on pBM600, the second of the indigenous plasmids essential for exosporium assembly in *B. megaterium*. Despite RT-PCR and (to a lesser extent) fluorescence microscopy analyses supporting the expression of this protein during sporulation, TEM analyses of *bxpB* null spores indicate that the protein has only a minor role in the assembly of the *B. megaterium* exosporium. Localization of the BclA1 protein in *bxpB* null spores also suggests that the mechanism of nap assembly in *B. megaterium* spores also differs to that observed in *B. cereus* family spores. The absence of additional BxpB orthologues encoded within the *B. megaterium* genome, and apparent differences in the time of optimal expression, confers further evidence that the nap proteins are anchored to the spore surface via a BxpB-independent mechanism. Indeed, bioinformatic analyses using known *B. cereus* family exosporium proteins as seeds revealed distinct orthologues of only three putative exosporium proteins encoded within the *B. megaterium* QM B1551 genome, indicating that the protein composition of the exosporia may differ substantially. The future challenge will be in identifying those essential plasmid-encoded protein(s) involved in nap localization and basal layer assembly in the *B. megaterium* exosporium.

## Supplementary Material

Supplementary data are available at FEMSLE online

## References

[bib1] Ball DA, Taylor R, Todd SJ (2008). Structure of the exosporium and sublayers of spores of the *Bacillus cereus* family revealed by electron crystallography. Mol Microbiol.

[bib2] Beaman TC, Pankratz HS, Gerhardt P (1972). Ultrastructure of the exosporium and underlying inclusions in spores of *Bacillus megaterium* strains. J Bacteriol.

[bib3] Eppinger M, Bunk B, Johns MA (2011). Genome sequences of the biotechnologically important *Bacillus megaterium* strains QM B1551 and DSM319. J Bacteriol.

[bib4] Gupta S, Ustok FI, Johnson CL (2013). Investigating the functional hierarchy of *Bacillus megaterium* PV361 spore germinant receptors. J Bacteriol.

[bib5] Henriques AO, Moran CP (2007). Structure, assembly, and function of the spore surface layers. Annu Rev Microbiol.

[bib6] Kailas L, Terry C, Abbott N (2011). Surface architecture of endospores of the *Bacillus cereus/anthracis/thuringiensis* family at the subnanometer scale. P Natl Acad Sci USA.

[bib9] McKenney PT, Driks A, Eichenberger P (2013). The *Bacillus subtilis* endospore: assembly and functions of the multilayered coat. Nat Rev Microbiol.

[bib10] McKenney PT, Driks A, Eskandarian HA (2010). A distance-weighted interaction map reveals a previously uncharacterized layer of the *Bacillus subtilis* spore coat. Curr Biol.

[bib8] McKenney PT, Eichenberger P (2012). Dynamics of spore coat morphogenesis in *Bacillus subtilis*. Mol Microbiol.

[bib7] Manetsberger J, Hall EA, Christie G (2014). BMQ_0737 encodes a novel protein crucial to the integrity of the outermost layers of *Bacillus megaterium* QM B1551 spores. FEMS Microbiol Lett.

[bib11] Ramirez-Peralta A, Gupta S, Butzin XY (2013). Identification of new proteins that modulate the germination of spores of *Bacillus* species. J Bacteriol.

[bib12] Redmond C, Baillie LW, Hibbs S (2004). Identification of proteins in the exosporium of *Bacillus anthracis*. Microbiology.

[bib13] Rosso ML, Vary PS (2005). Distribution of *Bacillus megaterium* QM B1551 plasmids among other *B. megaterium* strains and *Bacillus* species. Plasmid.

[bib14] Steichen CT, Kearney JF, Turnbough CL (2007). Non-uniform assembly of the *Bacillus anthracis* exosporium and a bottle cap model for spore germination and outgrowth. Mol Microbiol.

[bib15] Stevenson DM, Lach D, Vary PS, Balla E, Berencsie G (1993). A gene required for germination in *Bacillus megaterium* is plasmid-borne. DNA Transfer and Gene Expression in Microorganisms.

[bib16] Sylvestre P, Couture-Tosi E, Mock M (2002). A collagen-like surface glycoprotein is a structural component of the *Bacillus anthracis* exosporium. Mol Microbiol.

[bib17] Sylvestre P, Couture-Tosi E, Mock M (2005). Contribution of ExsFA and ExsFB proteins to the localization of BclA on the spore surface and to the stability of the *Bacillus anthracis* exosporium. J Bacteriol.

[bib18] Tan L, Turnbough CL (2010). Sequence motifs and proteolytic cleavage of the collagen-like glycoprotein BclA required for its attachment to the exosporium of *Bacillus anthracis*. J Bacteriol.

[bib19] Thompson BM, Stewart GC (2008). Targeting of the BclA and BclB proteins to the *Bacillus anthracis* spore surface. Mol Microbiol.

[bib20] Thompson BM, Hsieh HY, Spreng KA (2011). The co-dependence of BxpB/ExsFA and BclA for proper incorporation into the exosporium of *Bacillus anthracis*. Mol Microbiol.

[bib21] Todd SJ, Moir AJ, Johnson MJ (2003). Genes of *Bacillus cereus* and *Bacillus anthracis* encoding proteins of the exosporium. J Bacteriol.

