# Supplementary material for: Plasmid-encoded genes influence exosporium assembly and morphology in Bacillus megaterium QM B1551 spores
Source: FEMS Microbiol Lett. 2015 Aug 27;362(18):fnv147. doi: 10.1093/femsle/fnv147 (PMC4674009; doi:10.1093/femsle/fnv147)
Supplement: Supplementary data are available at FEMSLE online [file femsle_fnv147_index.html]

SUPPLEMENTARY DATA | FEMS Microbiology Letters

## SUPPLEMENTARY DATA

- SUPPLEMENTARY DATA
