# Supplementary material for: Plasmid-encoded genes influence exosporium assembly and morphology in Bacillus megaterium QM B1551 spores
Source: FEMS Microbiol Lett. 2015 Aug 27;362(18):fnv147. doi: 10.1093/femsle/fnv147 (PMC4674009; doi:10.1093/femsle/fnv147)
Supplement: Supplementary data are available at FEMSLE online [file Supplementary_Figures.docx]

Supplementary Figures

Figure S1





**Fig. S1** PCR-based assays to determine the plasmid composition of *B. megaterium* QM B1551 derived strains. Lane markers refer to plasmids pBM100 – pBM700 respectively. Oligonucleotides were designed to amplify plasmid-specific fragments from the following loci: (1) BMQ_pBM10002; (2) BMQ_pBM20007; (3) BMQ_pBM30024; (4) BMQ_pBM40024; (5) BMQ_pBM50077; (6) BMQ_pBM60048; (7) BMQ_pBM70070.

Figure S2


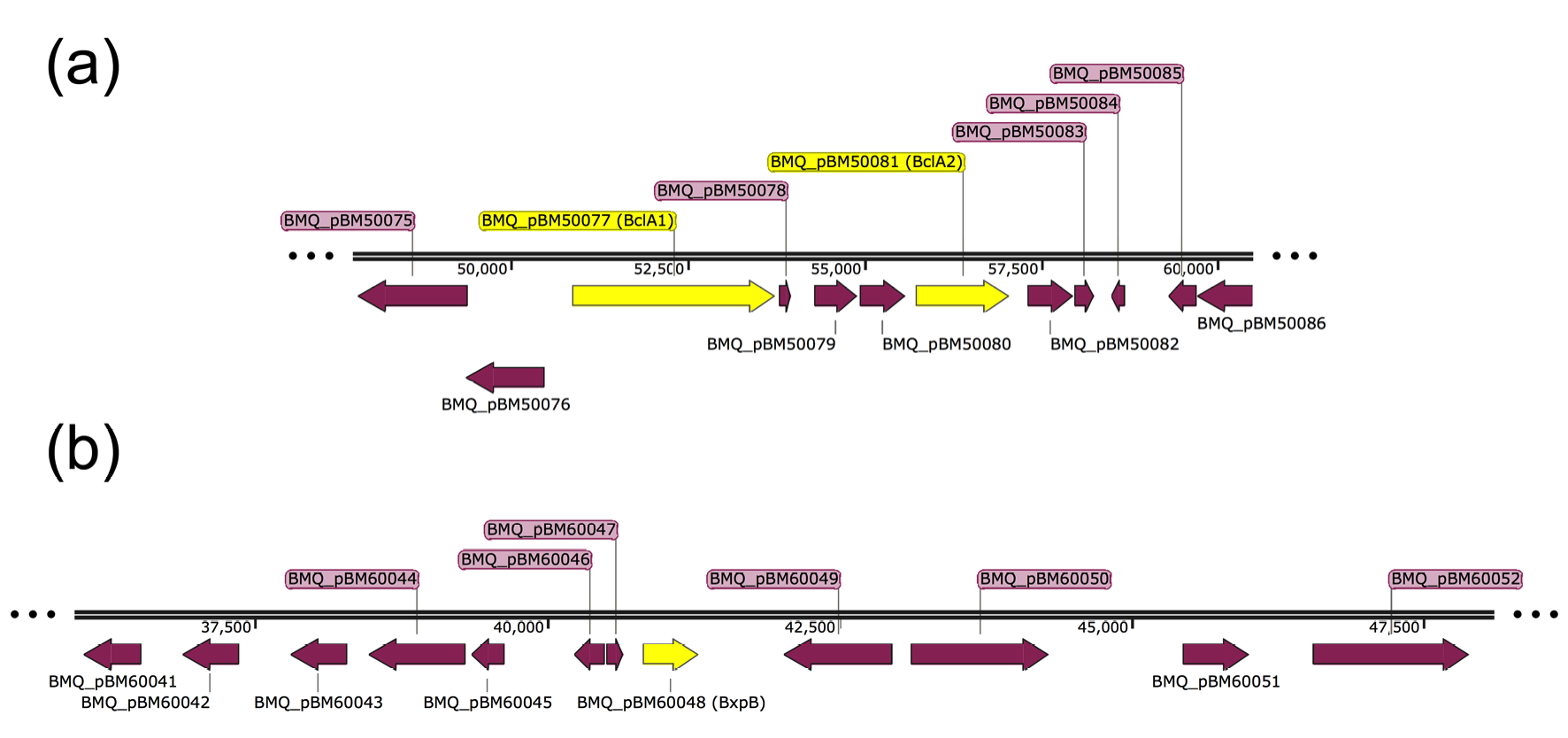


(c)

| Locus | Predicted protein |
| --- | --- |
| pBM500 loci |  |
| BMQ_pBM50075 | glycosyl transferase, group 2 family protein |
| BMQ_pBM50076 | glycosyl transferase, group 2 family protein |
| BMQ_pBM50077 | BclA1 |
| BMQ_pBM50078 | hypothetical protein |
| BMQ_pBM50079 | hypothetical protein |
| BMQ_pBM50080 | hypothetical protein |
| BMQ_pBM50081 | BclA2 |
| BMQ_pBM50082 | hypothetical protein |
| BMQ_pBM50083 | transcriptional regulator, MerR family |
| BMQ_pBM50084 | hypothetical protein |
| BMQ_pBM50085 | conserved domain protein |
| BMQ_pBM50086 | conserved hypothetical protein |
| pBM600 loci |  |
| BMQ_pBM60041 | hypothetical protein |
| BMQ_pBM60043 | hypothetical protein |
| BMQ_pBM60044 | integrase core domain protein |
| BMQ_pBM60045 | putative transposase |
| BMQ_pBM60046 | hypothetical protein |
| BMQ_pBM60047 | hypothetical protein |
| BMQ_pBM60048 | BxpB |
| BMQ_pBM60049 | YVTN family beta-propeller repeat family protein |
| BMQ_pBM60050 | transposase |
| BMQ_pBM60051 | conserved hypothetical protein |
| BMQ_pBM60052 | FAD binding domain protein |

**Fig. S2**. Approximate 10 kb regions from *B. megaterium* QM B1551 plasmids (a) pBM500, and (b) pBM600, showing the location of ORFs encoding BclA1, BclA2 and BxpB (all in yellow). Numbers refer to the plasmid position (in bp). Complete plasmid maps are shown in Eppinger *et al*., 2011. (C) Genbank annotations for loci depicted in (a) and (b).

Figure S3


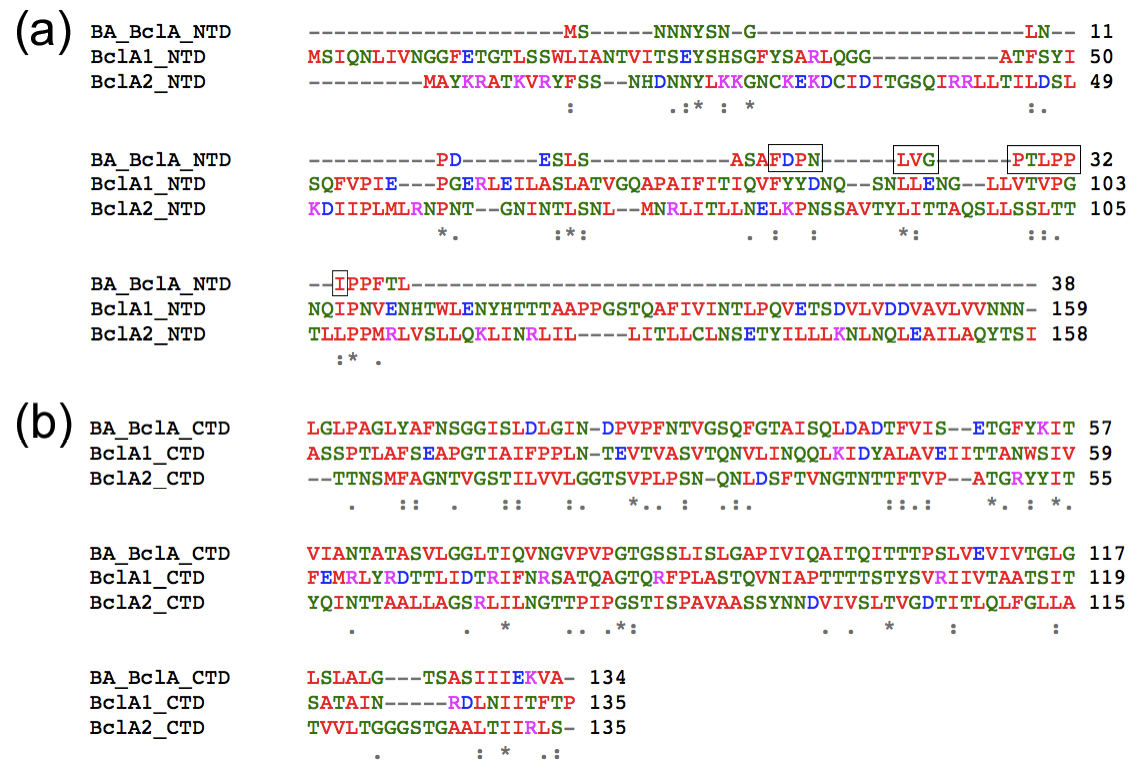


**Fig. S3** ClustalW alignment of (a) the N-terminal, and (b) C-terminal domains of BclA proteins from *B. anthracis* Sterne (BA_BclA) and *B. megaterium* (BclA1 and BclA2). The central collagen-like domains that are present in all three proteins are not included in the alignments. Boxed residues in (a) denote residues involved in targeting/anchoring BclA to the exosporium surface in *B. anthracis* Sterne (Tan & Turnbough Jr., 2010). Limited sequence conservation with this motif in the *B. megaterium* BclA proteins suggests that attachment to the exosporium may proceed via an alternative mechanism in spores of this species.

**Figure S4**


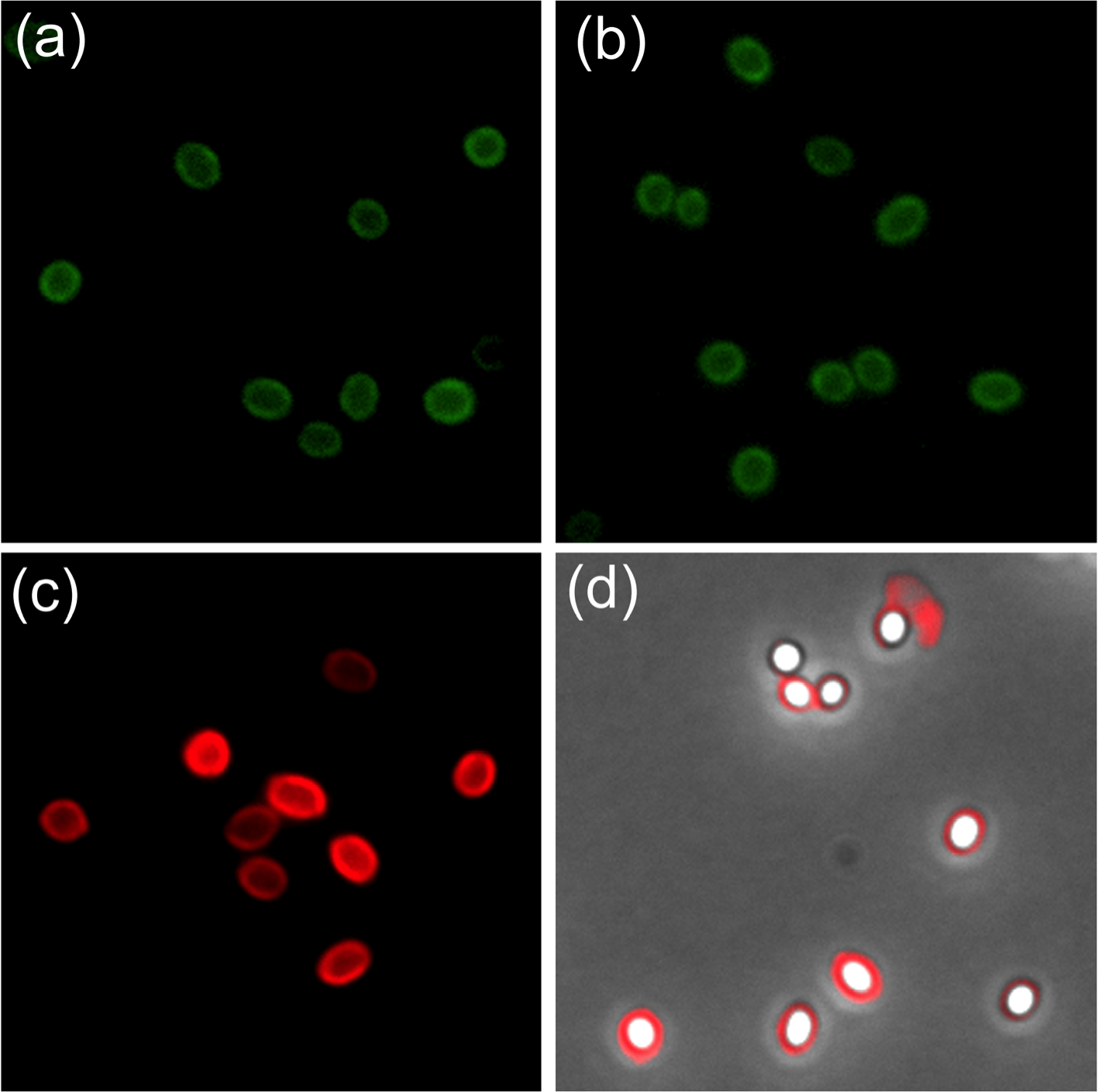


**Fig. S4** Fluorescence micrographs showing the localisation of putative inner-coat (SleL) and exosporium (CotX) proteins in *B. megaterium* QM B1551 wild-type and *bxpB* spores. (a) Wild-type *sleL*-*gfp* spores; (b) *bxpB* *sleL*-*gfp* spores; (c) wild type *cotX2*-*mCherry* spores; (d) *bxpB* *cotX2*-*mCherry* spores. The latter is an overlay of the fluorescent and phase images, showing the detached exosporium fragment in the uppermost spore.
